# Managing Patient-Generated Health Data Through Mobile Personal Health Records: Analysis of Usage Data

**DOI:** 10.2196/mhealth.9620

**Published:** 2018-04-09

**Authors:** Yu Rang Park, Yura Lee, Ji Young Kim, Jeonghoon Kim, Hae Reong Kim, Young-Hak Kim, Woo Sung Kim, Jae-Ho Lee

**Affiliations:** ^1^ Department of Biomedical Informatics Asan Medical Center Seoul Republic Of Korea; ^2^ Clinical Research Center Asan Medical Center Seoul Republic Of Korea; ^3^ Department of Convergence Medicine Asan Medical Center University of Ulsan College of Medicine Seoul Republic Of Korea; ^4^ Medical Information Office Asan Medical Center Seoul Republic Of Korea; ^5^ Department of Cardiology Asan Medical Center University of Ulsan College of Medicine Seoul Republic Of Korea; ^6^ Department of Pulmonary and Critical Care Medicine Asan Medical Center University of Ulsan College of Medicine Seoul Republic Of Korea; ^7^ Department of Emergency Medicine Asan Medical Center University of Ulsan College of Medicine Seoul Republic Of Korea

**Keywords:** personal health record, mobile health, patient engagement, patient-generated health data, health records, personal, telemedicine, patient participation

## Abstract

**Background:**

Personal health records (PHRs) and mHealth apps are considered essential tools for patient engagement. Mobile PHRs (mPHRs) can be a platform to integrate patient-generated health data (PGHD) and patients’ medical information. However, in previous studies, actual usage data and PGHD from mPHRs have not been able to adequately represent patient engagement.

**Objective:**

By analyzing 5 years’ PGHD from an mPHR system developed by a tertiary hospital in South Korea, we aimed to evaluate how PGHD were managed and identify issues in PGHD management based on actual usage data. Additionally, we analyzed how to improve patient engagement with mPHRs by analyzing the actively used services and long-term usage patterns.

**Methods:**

We gathered 5 years (December 2010 to December 2015) of log data from both hospital patients and general users of the app. We gathered data from users who entered PGHD on body weight, blood pressure (BP), blood glucose levels, 10-year cardiovascular disease (CVD) risk, metabolic syndrome risk, medication schedule, insulin, and allergy. We classified users according to whether they were patients or general users based on factors related to continuous use (≥28 days for weight, BP, and blood glucose, and ≥180 days for CVD and metabolic syndrome), and analyzed the patients’ characteristics. We compared PGHD entry counts and the proportion of continuous users for each PGHD by user type.

**Results:**

The total number of mPHR users was 18,265 (patients: n=16,729, 91.59%) with 3620 users having entered weight, followed by BP (n=1625), blood glucose (n=1374), CVD (n=764), metabolic syndrome (n=685), medication (n=252), insulin (n=72), and allergy (n=61). Of those 18,256 users, 3812 users had at least one PGHD measurement, of whom 175 used the PGHD functions continuously (patients: n=142, 81.14%); less than 1% of the users had used it for more than 4 years. Except for weight, BP, blood glucose, CVD, and metabolic syndrome, the number of PGHD records declined. General users’ continuous use of PGHD was significantly higher than that of patients in the blood glucose (*P*<.001) and BP (*P*=.03) functions. Continuous use of PGHD in health management (BP, blood glucose, and weight) was significantly greater among older users (*P*<.001) and men (*P*<.001). In health management (BP, weight, and blood glucose), overall chronic disease and continuous use of PGHD were not statistically related (*P*=.08), but diabetes (*P*<.001) and cerebrovascular diseases (*P*=.03) were significant.

**Conclusions:**

Although a small portion of users managed PGHD continuously, PGHD has the potential to be useful in monitoring patient health. To realize the potential, specific groups of continuous users must be identified, and the PGHD service must target them. Further evaluations for the clinical application of PGHD, feedback regarding user interfaces, and connections with wearable devices are needed.

## Introduction

Patient centeredness and patient engagement are essential characteristics of health care services and provide the greatest benefits to patients [1]. In precision medicine, patient engagement and patient-generated health data (PGHD) are regarded to be as important as clinical and genomic data [2-5]. As wired, widespread tools for data collection, mobile phones and apps can generate engagement and gather data [2,3,5,6]. Mobile patient health records (mPHRs) can integrate and manage such kinds of data and can be connected with other mobile services. Moreover, for personalized care and customized treatment, sufficient patient data are required [6,7]; intermittent information collected at the hospital may not provide sufficient patient data [8]. Therefore, the establishment of a health platform for patients and patient participation is needed, and patient health records (PHRs) are an appropriate tool for this purpose [6].

Patient information accumulated through mPHRs and wearable devices can help build a personalized baseline [2]. PGHD have the potential to change the paradigm for existing normal ranges [2]. Information gathering through patient health platforms is expected to benefit medical care and patient health outcomes [9-14]. Meanwhile, there are concerns about the construction of such patient health platforms [10,15,16]. In particular, mHealth apps, which are easily accessible to patients and health personnel, are frequently discontinued and discarded [17]. It is necessary to encourage long-term use to maximize the effects of the health outcomes of the health platform and fully utilize the collected information. To this end, it is necessary to analyze factors that affect the long-term use of health platforms. Several studies have been conducted regarding this topic.

Previous studies have noted the lack of usage data research that analyzes the use of mPHRs from the perspective of PGHD [6,10]. In particular, there is a lack of research on modifiable factors (eg, service menu) and the persistence of health platforms based on data. In addition to user-specific characteristics (eg, age, sex, diagnosis), studies should be conducted on the modifiable factors that affect use duration, to facilitate activities that promote continued use.

We conducted this study using data on the 5-year use of an mPHR system distributed by a tertiary hospital in South Korea. The mPHR system, which has been used since 2010, provides several functions through which users can log their health data. Based on actual usage data, we investigated the usage pattern and characteristics of the users of PGHD services. To the best of our knowledge, this is the first study on the long-term use and input of PGHD through mPHRs.

## Methods

### Data and Mobile Patient Health Record Description

We collected the log data of an Android-based mPHR app called My Chart in My Hand (MCMH) at Asan Medical Center (AMC), which is the largest general hospital in South Korea. AMC established the Ubiquitous Health Center in 2009, and the MCMH was implemented on December 27, 2010, after collaboration with a Korean telecommunication company (SK Telecom Co Ltd, Seoul, Republic of Korea) [18]. The Ubiquitous Health Center is responsible for the development, operation, and management of telehealth services and various apps related to mHealth in AMC. Released in January 2011, MCMH is the first mPHR in South Korea; it enables patients to view and manage their own health records [19,20]. MCMH 2.0 has been operational since 2016; it offers more diverse functions for patient engagement (disease diaries and assessment tools [patient survey] for symptoms, lifestyle, quality of life, and stress, which can be used in clinics for cancer, inflammatory bowel disease, diabetes, and pediatric asthma and atopy) and medication consultations with a clinical pharmacist. This study is a user pattern analysis for MCMH 1.0, which was operational from the end of 2010 until 2015. MCMH 1.0 provides the following 4 menus: My chart, Health management, Medication management, and Outpatient support service [21]. Among these 4 functions, PGHD belong to the Health management, Medication management, and My chart menus. MCMH is not restricted to AMC patients. General users can download the app and use the functions related to the above PGHD, although the functions connected to the AMC hospital information system are limited to its patients.

**Figure 1:**
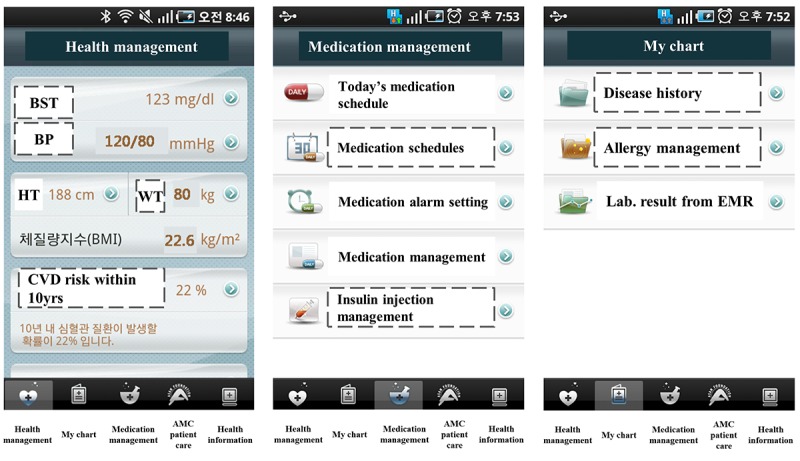
Patient-generated health data (PGHD) screens in the My Chart in My Hand app for the Health management, Medication management, and My chart menus. The functions corresponding to PGHD in the 3 screens are indicated by dashed boxes. The original app showed menu names in Korean, which have been translated into English. AMC: Asan Medical Center; BMI: body mass index; BP: blood pressure; BST: blood glucose level; CVD: cardiovascular disease; HT: height; EMR: electronic medical record; WT: weight.

The items in dashed boxes in Figure 1 show the detailed PGHD items that the user stores in MCMH. The health management function provides features for tracking and updating PHRs, such as blood glucose levels, blood pressure (BP), and weight. Based on the information entered by patients, body mass index, 10-year cardiovascular disease (CVD) risk, and metabolic syndrome risk can be calculated. The medication management function provides medication schedulers and reminders of when to take medicines. Users can manage their medication schedules (Medication) and insulin injections (Insulin) themselves through this function. Users are required to input data manually on these PGHD functions, as there is no functionality for accepting data streams from personal tracking devices.

### Study Design

To identify the usage pattern of the PGHD functions according to the type of PGHD, user type (patient or general user), and continuous use of the function, we analyzed the logs of all users who signed up and logged in more than once between December 2010 and December 2015. MCMH 1.0 was launched on December 20, 2010, for test users and on December 27, 2010 for all users. It was replaced with MCMH 2.0 on December 31, 2015.

As there are no existing criteria for continuous use of PGHD services, this study defined the criteria for each PGHD function. We defined continuous use as follows: weight, BP, and blood glucose entered at least once per week and used for at least 4 weeks (28 days); 10-year CVD risk and metabolic syndrome risk entered at least twice and used for at least 180 days. Because weight, BP, and blood glucose are continuous values that indicate users’ daily health status, 10-year CVD risk or metabolic syndrome risk is a risk-evaluation function that has no definite consensus regarding the evaluation period; we derived these criteria differently through discussion.

The user logs contained time stamps for each PGHD function, recorded whenever an individual used these functions. We also gathered demographics and medical records for patients, such as age, sex, residence, and health information, including hospital visits and presence of chronic diseases, using our clinical research data warehouse [22]. We conducted demographic and medical record comparison analysis of patients between continuous use and noncontinuous use by classifying the PGHD variables into health (BP, weight, and blood glucose) and risk (10-year CVD risk and metabolic syndrome risk) management. Distance from AMC to the patient’s residence was designated as short distance if the patient lived in the capital region with AMC and as long distance if the patient lived outside the capital [18]. The presence of chronic disease was classified by the definitions of the Korea Center for Disease Control and Prevention: cancer (C00-C97), diabetes (E10-E14), CVD (I20-I51), cerebrovascular disease (I60-I69), chronic lower respiratory disease (J40-J47), and liver disease (K70-K76) [23,24]. We classified all diseases according to the *International Classification of Diseases, 10th Revision*.

This study was approved by the AMC’s institutional review board (no. 2017-1128). The ethics committee waived the need for informed consent, as this study used routinely collected log data that were anonymously managed at all stages, including during data cleaning and statistical analyses.

**Figure 2:**
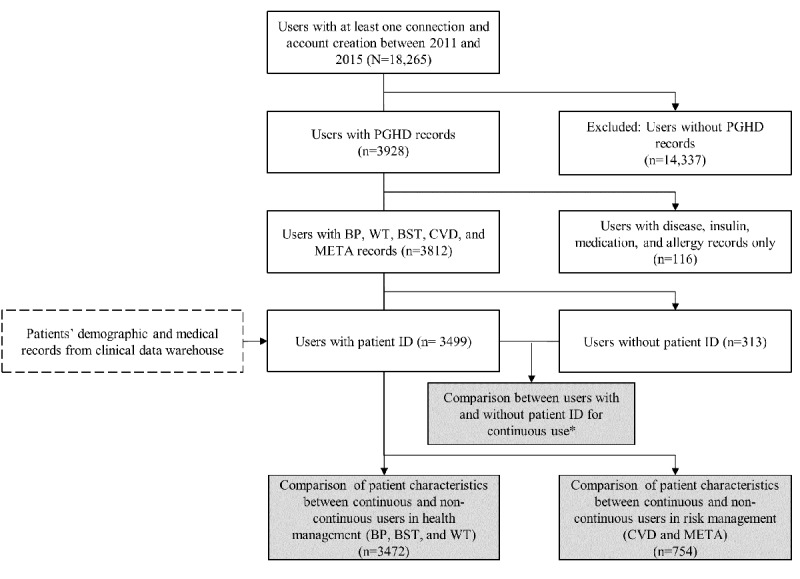
Patient inclusion and exclusion criteria (white boxes) and flow through the study. The gray boxes show user log analyses. The dashed boxes indicate additional patient clinical data obtained from the users. *Criteria for continuous use: weight (WT), blood pressure (BP), and blood glucose level (BST) entered at least once per week and used for at least 28 days; 10-year cardiovascular disease (CVD) risk and metabolic syndrome (META) risk entered at least twice and used for at least 180 days. ID: identifier; PGHD: patient-generated health data.

### Data Analysis

Figure 2 shows the patient selection flow for the study. Among a total of 162,661 users who downloaded and created an MCMH account, we excluded 144,396 users who had never accessed MCMH services. Therefore, we considered a total of 18,265 actual MCMH users for inclusion in the study. We first excluded 14,337 users without PGHD records. The number of those with PGHD records who used only the disease, insulin, medication, and allergy functions was relatively small, with 116 users, so we focused on 3812 users with records for BP, weight, blood glucose, 10-year CVD risk, and metabolic syndrome.

We performed a comparative analysis of the continuous use of PGHD services between AMC patients (n=3499) and general app users (n=313). To analyze the demographic and clinical characteristics, we extracted related variables from the clinical data warehouse only for patients. We then analyzed patients’ characteristics according to their continuous use of health management (BP, weight, and blood glucose) and risk management (10-year CVD risk and metabolic syndrome risk) functions.

We compared means and of frequencies with the Student *t* test and chi-square test, respectively. If the observed value was less than 5, we performed Fisher exact test. All reported *P* values were 2-sided, and *P* values less than .05 were considered significant. Also, we performed a multiple logistic regression analysis with adjusted age and sex. Data analyses were conducted with the R software, version 3.3.1 (R Foundation).

## Results

### Overall Use Characteristics

Within 5 years of MCMH operation, a total of 18,265 users downloaded the app and logged in more than once. Among these users, 16,729, or 91.59% of the total, were AMC patients. Patients had a statistically significant (1.8 times) longer use of the system (average period of use: 251.86 vs 467.84 days) and a pattern of accessing the app more than 6 times compared with general users (average number of accesses: 8.26 vs 50.96).

Among all users, 21.50% (3928) had at least one PGHD record (Table 1). In the PGHD, data input was significantly higher in the group of general users: blood glucose (*P*=.003), weight (*P*<.001), and allergy (*P*=.04). The average number of records per user was also significantly higher in general users: 6.89 (SD 24.79) vs 4.41 (SD 31.46) (*P*=.009). The median values of 1 for all categories except insulin indicate that more than half of the users made only 1 entry in each category. There was no significant difference in the remaining PGHD items.

**Table 1:** Numbers of users who entered patient-generated health data in the app by user type (hospital patients and general app users).

Variables	General users (n=329)			Patients (n=3599)			Total (n=3928)			*P* value^a^
	No.	Median	Mean (SD)	No.	Median	Mean (SD)	No.	Median	Mean (SD)	
Blood pressure	143	1	9.13 (27.31)	1482	1	5.87 (43.56)	1625	1	6.16 (42.39)	.10
Weight	275	1	1.44 (2.17)	3345	1	2.71 (15.89)	3620	1	2.61 (15.29)	<.001
Blood glucose level	154	1	20.39 (45.03)	1220	1	9.45 (51.92)	1374	1	10.70 (51.31)	.003
10-year cardiovascular disease risk	81	1	1.29 (1.04)	683	1	1.30 (1.24)	764	1	1.29 (1.22)	.49
Metabolic syndrome risk	75	1	1.32 (1.19)	610	1	1.26 (1.05)	685	1	1.27 (1.07)	.36
Medication	44	1	2.25 (2.79)	208	1	2.50 (3.82)	252	1	2.46 (3.66)	.303
Disease	60	1	1.58 (1.40)	115	1	1.45 (0.85)	175	1	1.49 (1.07)	.26
Insulin	16	1	15 (31.94)	56	2	6 (10.11)	72	2	8.02 (17.90)	.15
Allergy	16	1	1.18 (0.52)	45	1	1.88 (2.52)	61	1	1.70 (2.20)	.04

^a^Student *t* test.

**Figure 3:**
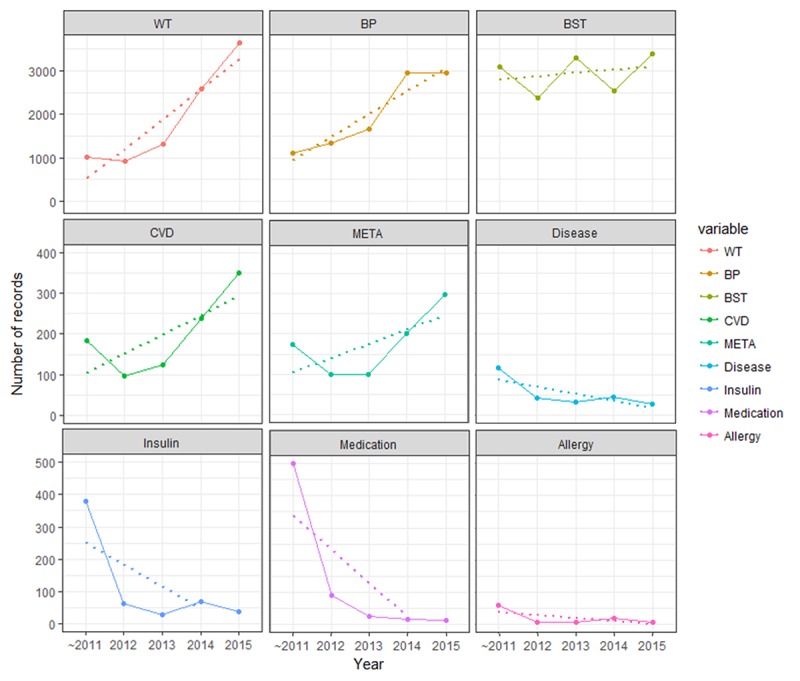
Analysis of the tendency of patient-generated health data (PGHD) to increase or decrease by year. The mobile patient health record log of input by type of PGHD was analyzed by year. The solid line represents the actual number of records, and the dashed line represents the trend for the record. BP: blood pressure; BST: blood glucose level; CVD: 10-year cardiovascular disease risk; META: metabolic disease risk; WT: weight.

### Patient-Generated Health Data Entry Distribution

The distribution of total PGHD items was divided into 2 patterns: increasing and decreasing (Figure 3). Items with an increasing pattern were Health management menu items: weight, HT, BP, blood glucose, 10-year CVD risk, and metabolic syndrome risk. Items that showed a decreasing pattern were diseases, insulin, medication, and allergy, belonging to the Medication management and My Chart menus. Among the increasing patterns, the weight value increased the fastest (slope-685.8, *R*^2^=.857), followed by BP (slope=526.1, *R*^2^=.884), and 10-year CVD risk (slope=47.2, *R*^2^=.552). In the decreasing pattern, medication showed the steepest decrease (slope=–104.6, *R*^2^=.623).

**Figure 4:**
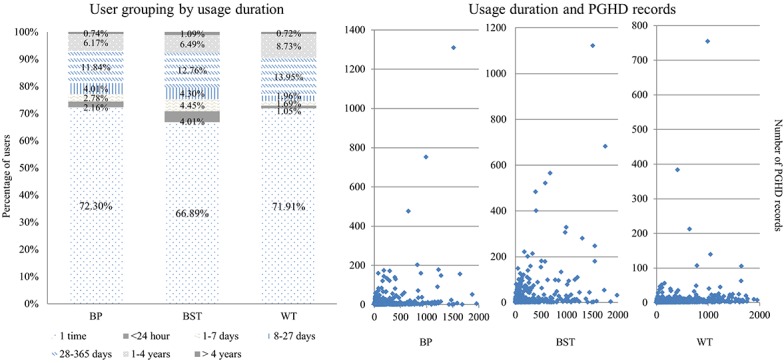
Analysis of patient-generated health data (PGHD) by duration of app use. Left: Duration of use divided into 7 categories, from once to more than 4 years of use. Right: Actual duration of use (x-axis) and number of PGHD records generated by each user (y-axis); 1 point represents 1 user. BP: blood pressure; BST: blood glucose level; WT: weight.

We performed a periodic usage analysis of the 3 most recorded PGHD items (BP, weight, and blood glucose) among the 9 PGHD items (Figure 4). To determine how long users took to enter their PGHD, we first divided the users into 7 groups based on the duration of use (Figure 4). According to the analysis, approximately 70% of users generated PGHD only once; 11% to 14% of them used MCMH for more than 4 weeks but less than 1 year, and only 6% to 9% used it for more than 1 year and less than 4 years. Only less than 1% of users used it for more than 4 years. In the graph between the actual value of usage duration and PGHD records (Figure 4), some users have PGHD records for more than 5 years (over 1825 days), which is the result of transferring users’ preexisting records before the MCMH service started.

### Comparison Between Patients and General Users in Continuous Use of Patient-Generated Health Data Functions

To characterize continuous users of the PGHD functions, we defined the criteria for continuous use of health (at least 28 days for BP, weight, and blood glucose) and risk (at least 180 days for 10-year CVD risk and metabolic syndrome risk) management, and then analyzed the differences between AMC patients and general app users. A total of 175 mPHR users continued to use the PGHD functions. General users were significantly higher than patients in continuous use of PGHD for blood glucose (*P*<.001) and BP (*P*=.03). For other PGHD items, there was no statistically significant difference in continuous use between the 2 user types (Table 2).

### Characteristics of Patients Who Continuously Used Patient-Generated Health Data Functions

To identify the characteristics of users who continuously used PGHD functions, we conducted a comparative analysis of the related data on demographics, diagnoses, and hospital visits in the health and risk management menus of MCMH (Table 3). This analysis was limited to patients, for whom demographic, diagnostic, and hospital visit records could be identified. A total of 142 patients used PGHD continuously. The continuous use of PGHD services in the health management sector was statistically significant for older individuals and men (both *P*<.001). For continuous use of PGHD services, there was no statistically significant difference in overall chronic disease (*P*=.08), but diabetes (*P*<.001) and cerebrovascular diseases (*P*=.03) differed significantly. These characteristics were also significant in age- and sex-adjusted multivariate regression analyses (diabetes: *P*<.001; cerebrovascular disease: *P*=.03). In those with diabetes, continuous users were younger than noncontinuous users (average age 42.75 vs 49.50 years, *P*=.04). This is young relative to the average age of continuous users of the entire PGHD group (average age 42.75 vs 51.81 years, respectively). In those with cerebrovascular disease, continuous users were older than noncontinuous users, which was not statically significant (average age 61 vs 48.2 years, *P*=.33). In each disease group, there was no significant difference between the 2 groups (continuous vs noncontinuous) in sex, distance from the hospital, and type of visit.

Hospital visit experience was not statistically related to continuous use; all continuous users made emergency room and outpatient visits. In risk management, there were no significant differences between continuous and noncontinuous users.

**Table 2:** Comparison between general app users and Asan Medical Center patients for continuous use of patient-generated health data functions.

Categories/Variables/Continuous use			Users, n (%)			*P* value^a^
			General users (n=313)	Patients (n=3499)	Total (n=3812)	
**Health management**						
	**Blood pressure (n=1621)**					.03
		No	130 (8.30)	1437 (91.70)	1567	
		Yes	9 (16.67)	45 (83.33)	54	
	**Weight (n=3620)**					.70
		No	275 (7.64)	3323 (92.36)	3598	
		Yes	0 (0.0)	22 (100.00)	22	
	**Blood glucose level (n=1371)**					<.001
		No	128 (9.98)	1154 (90.02)	1282	
		Yes	23 (25.84)	66 (74.16)	89	
**Risk management**						
	**10-year cardiovascular disease risk (n=764)**					.97
		No	76 (10.61)	640 (89.39)	716	
		Yes	5 (10.42)	43 (89.58)	48	
	**Metabolic syndrome risk (n=685)**					.72
		No	71 (11.06)	571 (88.94)	642	
		Yes	4 (9.30)	39 (90.70)	43	

^a^Chi-square test.

**Table 3:** Characteristics of patients who used patient-generated health data functions in health and risk management continuously (“Yes”) versus those who did not (“No”).

Variables/Categories		Health management^a^ (n=3472)				Risk management^b^ (n=754)			
		Yes (n=94)	No (n=3378)	*P* value^c^	*P* value^d^	Yes (n=50)	No (n=704)	*P* value^c^	*P* value^d^
Age (years), mean (SD)		51.81 (12.07)	43.79 (15.37)	<.001	N/A^e^	46.98 (11.67)	47.92 (11.24)	.58	N/A
**Sex, n (%)**				<.001	N/A			.75	N/A
	Male	76 (81)	2101 (62.20)			35 (70)	470 (66.8)		
	Female	18 (19)	1277 (37.80)			15 (30)	234 (33.2)		
**Distance to the hospital, n (%)**				.41	N/A			.26	N/A
	Short	35 (37)	1103 (32.65)			12 (24)	231 (32.8)		
	Long	59 (63)	2275 (67.34)			38 (76)	473 (67.2)		
**Disease classification^f^, n (%)**		
	Cancer (C00-C97)	11 (12)	606 (17.94)	.14	.08	8 (16)	99 (14.1)	.87	.63
	Diabetes (E10-E14)	12 (13)	122 (3.61)	<.001	<.001	2 (4)	29 (4.1)	>.99	.96
	Cardiovascular disease (I20-I51)	4 (4)	90 (2.66)	.33	.55	2 (4)	19 (2.7)	.64	.57
	Cerebrovascular disease (I60-I69)	4 (4)	38 (1.12)	.03	.03	0 (0)	14 (2.0)	.62	.98
	Chronic lower respiratory disease (J40-J47)	1 (1)	15 (0.44)	.36	.35	0 (0)	2 (0.3)	>.99	.98
	Liver disease (K70-K76)	14 (15)	299 (8.85)	.08	.42	4 (8)	72 (10.2)	.79	.59
	Chronic disease	39 (42)	1070 (31.68)	.08	.27	15 (30)	218 (31.0)	>.99	.92
**Type of hospital visit, n (%)**		
	Emergency room	59 (62)	1811 (53.36)	.15	N/A	30 (59)	371 (52.1)	.43	N/A
	Outpatient department	94 (98)	3,334 (98.70)	.68	N/A	49 (96)	699 (98.2)	.264	N/A
	Hospitalization	76 (79)	2,689 (79.60)	>.99	N/A	42 (82)	544 (76.4)	.423	N/A

^a^Blood pressure, weight, and blood glucose.

^b^Metabolic syndrome and 10-year risk of cardiovascular disease.

^c^*t* test (for continuous), chi-square test, or Fisher exact (for categorical) test.

^d^Multiple logistic regression test adjusted for age and sex.

^e^N/A: not applicable.

^f^Korea Center for Disease Control and Prevention classifications.

## Discussion

### Principal Findings

The mPHR used in this study was a feasible platform for managing PGHD, for the following reasons. First, it was available not only to patients but also to general users; it was used as a platform to store and refer to general users’ health information. Although the number of users was small, the fact that there were long-term users entering their health information means that this app was used as a suitable tool for storing and referring to health information. Second, there were enough long-term users to show significant differences in usage patterns. Third, the upgraded version, which reflects usage patterns and user needs, of the existing mPHR is expected to improve user satisfaction and to contribute additional data for further research.

This study is unique compared with previous studies based on the following characteristics. First, we used actual usage data to investigate long-term use. Meanwhile, research on telecare for chronic disease management lacks enough studies for a sufficient period of time. Second, our analysis was based on users rather than just on patients of AMC. We also compared usage by those who had been in hospital (patients) and those who had not (general users). Third, the mPHR we examined is the first one to provide patient information to patients in Korea, and our study included analysis and consideration of the modifiable factors for long-term use promotion.

### Overall Usage Pattern and User Characteristics

Most of the mPHR users were patients (91.59%), and we found them to have significantly longer and more frequent use. However, general users used more of the overall PGHD functions (*P*=.009). There was a significant difference in blood glucose (*P*<.001). General users submitted more BP and insulin records, although the differences were not significant. MCMH is not a type of mPHR that mainly aims at chronic disease management; it is a comprehensive platform that includes such services as providing patient information and setting appointments. Therefore, the more frequent use by patients is attributed to the greater number of services available through the mPHR relevant to them than to general users. Nonetheless, general users used it more actively for storing and referring to health information. This result confirms the potency for managing patient health information through an mPHR.

### Patient-Generated Health Data Entry Distribution

According to a 2011 survey by the Consumer Health Information Corporation, 26% of health apps were abandoned after one use, whereas 79% were used up to 10 times before being abandoned [25]. Approximately 70% of the users of the mPHR entered PGHD only once, but this should be considered in the context that PGHD is one of the functions of the mPHR. In addition, about 10% of PGHD users (weight: 9.45%; blood glucose: 7.49%; BP: 6.91%) entered health records for more than 1 year. Thus, the PGHD functions have the potential for long-term health monitoring.

The total use of weight, BP, blood glucose, CVD, and metabolic syndrome menus tended to increase, whereas the use of diseases, insulin, medication, and allergy menus tended to decline. The data to be input into the diseases, insulin, medication, and allergy menus (eg, entering the whole name and dose of the medication and selecting the insulin injection site) are more complicated than in other functions. On the other hand, BP, weight, and blood glucose menus required users to input only a few numerical values, and CVD and metabolic syndrome required users to check several boxes for risk evaluation. The user interface problem at the time of data input can be considered to have caused these differences. In addition, while biosignals such as weight, BP, and blood glucose were entered for the purpose of managing health data by the users themselves, the functions that are out of the scope of the users’ management might provide less motivation for continuous use, without rewards such as the feedback of clinicians. Also, the increase in the use of biosignal input functions suggests that automatic input of data through wearable devices, body scales, or blood glucose meters may be helpful for encouraging continuous use. Linking PHR data would reduce the inconvenience to users of inputting data for diseases, insulin, medication, and allergy.

### Comparison Between Patients and General Users

General users’ tendency to use the blood glucose and insulin functions longer showed the need for a reliable app service for diabetes management. The mPHR was developed for general care, not only for chronic disease management. Therefore, services for patients with chronic diseases were limited. However, despite the small proportion, there were long-term users for chronic diseases management, especially for diabetes.

One of the main reasons for the abandonment of health apps is the mistrust of app developers [17]. Although many studies have indicated the effectiveness of diabetes management through patient health platforms, the use of mobile apps for diabetes management was not universal in Korea at the time of the MCMH mPHR launch. Recently, PHRs focused on chronic diseases have emerged [26]. Therefore, users looking for a reliable app for diabetes management, even though they were not patients of AMC, used the MCMH mPHR.

### Patients’ User Characteristics

Significantly, male users, elderly users (mean age 51.81 vs 43.81 years), and users with a diagnosis of diabetes tended to use the health management functions continuously. In the case of cancer, which accounted for more than half of all chronic diseases, the incidence in Korea increased by age beginning with users in their 80s, and the incidence among men was higher than that among women (445.2/100,000 vs 397.6/100,000 [27]). In addition, according to a 2015 report in Korea, the average age at first diagnosis of diabetes mellitus was 57.11 (SD 13.9) years for men and 60.57 (SD 14.9) years for women. According to a survey in 2016, in South Korea, the percentage of males who own a smartphone is higher than that of females, and the percentage decreases after the age of 30 years [28]. Nonetheless, the high proportion of long-term use of PGHD services by elderly patients reflects the age characteristics of patients living with chronic diseases. However, in a subanalysis of users with a diagnosis of diabetes, the younger users (average ag: 42.75 vs 49.50 years) tended to use PGHD services continuously, and there was no significant difference in the sex of users. Hence, the number of continuous users with diabetes was small (n=12), and more in-depth research such as user surveys or interviews is required to understand the detailed usage patterns.

### Limitations of This Research

The main limitation of this study was the lack of a clinical practice application of the PGHD collected in the mPHR; the PGHD in MCMH version 1.0 were used for simple reference without any feedback from a health provider. This aspect is improved in MCMH 2.0, and the PGHD in the mPHR are used clinically in centers for diabetes, cancer, inflammatory bowel diseases, and pediatric and atopic asthma.

Another limitation was the definition of continuous use of PGHD services. In this study, we defined our own criteria through discussions among the researchers. Various criteria may be applied when considering the nature of patients’ diseases and hospital visit intervals. The low percentage of those who used the service continuously was also a major drawback of this analysis.

Ease of data entry can also affect PGHD service usage. Since wearable devices were not connected to our mPHR, we expected that data input convenience would be poor. Therefore, if wearable devices could be linked to the mPHR, the low compliance may be improved. However, appropriate devices and scenarios need to be considered to collect PGHD effectively. Previously, encouraging results have been reported in cancer and diabetes management. A recent study focused on short-term patient management through wearable devices [29]. Further research on collecting PGHD through wearable devices and long-term mPHR operation in clinical applications should be conducted. In this study, we found a relatively small number of users who continuously used the mPHR’s PGHD functions. There are many possible causes for this (eg, user interface inconvenience, low motivation, input error), but further research such as conducting questionnaire surveys is also necessary for clearer understanding.

### Conclusion

Although a small proportion of users managed their PGHD input continuously through the mPHR, we found the mPHR to be a tool for integrating PGHD and patient medical information. Studies examining the factors promoting the continuous use of PGHD functions in mPHRs and the consensus of the continuous use of various PGHD types are needed. Further evaluation for the clinical application of PGHD, feedback regarding user interfaces, and connections with wearable devices are needed as well.

## Abbreviations

AMC: Asan Medical Center
BP: blood pressure
CVD: cardiovascular disease
MCMH: My Chart in My Hand
mPHR: mobile personal health record
PGHD: patient-generated health data
PHR: personal health record
